# The mitochondrial genome of the planorbid snail *Planorbella duryi*

**DOI:** 10.1080/23802359.2018.1503939

**Published:** 2018-10-30

**Authors:** Jonathan H. Schultz, Lauren M. Bansbach, Jarrett A. Bremmer, Kirsten E. Dimmler, Quinn A. Forde, Elisa M. Gagliano, Elizabeth M. Glenn, Chance M. Greengrass, Joe P. Hayes, Aurora L. Kraus, Lewis I. Larsen, Erin Lucero, Matthew T. McClendon, Heather L. Mercer, Karen C. Mims, Kajal N. Patel, Fotios I. Patsalis, Dianne E. Peterson, Jarrod M. Platero, Mohammed M. Rizvi, Kassandra I. Serna, Tyler E. Steele, Nicholas L. Turner, Lijing Bu, Lijun Lu, Coen M. Adema

**Affiliations:** Department of Biology, 1 University of New Mexico, Albuquerque, NM, USA

**Keywords:** Gastropoda, Planorbidae, mitogenome, Mollusca

## Abstract

The complete mitochondrial genome of a freshwater planorbid snail, *Planorbella duryi* (Mollusca, Gastropoda) was recovered from *de novo* assembly of genomic sequences generated with the Illumina NextSeq500 platform. The *P. duryi* mitogenome (14,217 base pairs) is AT rich (72.69%) and comprises 13 protein-coding genes, two ribosomal subunit genes, and 22 transfer RNAs. The gene order is identical to that of *Biomphalaria glabrata* and other snail species in the family Planorbidae. This is the first full characterization of a mitochondrial genome of the genus *Planorbella.*

## Report

Mitogenome data have benefited phylogenetic reconstructions of the class Gastropoda (phylum Mollusca), a group with a complex taxonomy (White et al. 2011; Williams et al. 2014; Oskars et al. 2015). This report presents the mitochondrial genome for the snail *Planorbella duryi* adding to the limited availability of mitogenome data available for the family Planorbidae that includes several medically important snails that transmit schistosome parasites of humans. (DeJong et al. 2004; Jannotti-Passos et al. 2010; Zhang et al. 2018).

An individual snail, morphologically identified as a planorbid, was collected from Shady lakes trout fishing resort in Albuquerque, NM (35.2144° N, 106.5956° W). After removal of the shell, genomic DNA was extracted from whole-body tissues using a CTAB-based method (Winnepenninckx et al. 1993). High-throughput DNA sequencing was carried out using the KAPA Hyper Prep Kit, Illumina^®^ platforms (KAPPA Biosystems) and the Illumina NextSeq500 at the Molecular Biology Facility of UNM. The whole body tissues of *P. duryi* were destructively sampled for DNA, however, all genomic Illumina data were deposited in the SRA database (GenBank accession SRP151592). The mitochondrial genome and two nuclear genes (18S, 28S) were assembled using MITObim v 1.8 (Hahn et al. 2013). MITOS (Bernt et al. 2013) annotation of the assembled mitogenome was manually checked to minimize overlaps of protein-coding genes and identify candidate stop codons that are completed by polyadenylation (DeJong et al. 2004). Protein-coding (amino acid) sequences of complete mitogenomes of *P. duryi* (KY514384) and select gastropod taxa were aligned using ClustalX (Larkin et al. 2007) and trimmed to minimize gaps at sequence termini. Phylogenetic reconstruction was performed using MEGA v.7 (Kumar et al. 2016) and Neighbor-Joining statistical method with 1,000 bootstrap replicates (JTT model with gamma distributed rate variation among sites).

**Figure 1.**
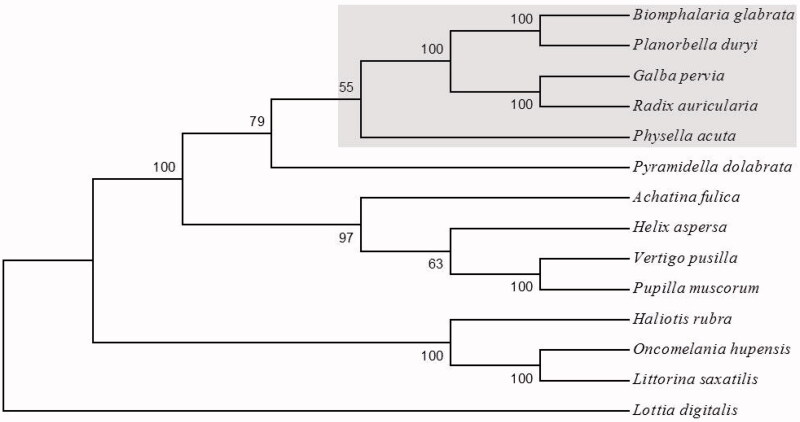
Neighbor-joining tree of complete mitogenomes (protein-coding sequences) of Planorbella duryi and 13 gastropod species. *Planorbella duryi* is placed among species of the taxonomic clade Hygrophila (shaded). The basal gastropod Lottia digitalis is used as an outgroup. Accessions for full mitogenome sequences: *Achatina fulica* (NC_024601), *Biomphalaria glabrata* (NC_005439), *Galba pervia* (NC_018536), *Haliotis rubra* (NC_005940), *Helix aspersa* (NC_021747), *Littorina saxatilis* (NC_030595), *Lottia digitalis* (NC_007782), *Oncomelania hupensis* (NC_013073), *Physella acuta* (NC_023253), *Planorbella duryi* (KY514384), *Pupilla muscorum* (NC_026044), *Pyramidella dolabrata* (NC_012435), *Radix auricularia* (NC_026538), *Vertigo pusilla* (NC_026045).

The species designation *Planorbella duryi* was consistent with snail morphology, knowledge of local snail fauna, and BLAST analyses of 18S and 28S sequences (GenBank accessions KY514382 and KY514383). The complete mitochondrial genome of *P. duryi* is 14,217 bp in length (GenBank accession: KY514384) and contains 13 protein-coding genes, two ribosomal subunit genes (12S, 16S), and 22 tRNAs. Similar to other gastropod species, the *P. duryi* mitogenome is AT rich with a base composition of 31.01% A, 13.03% C, 14.29% G, and 41.68% T. Illustrative of typical panpulmonate snails (excluding *Physella acuta*; Nolan et al. 2014), the gene order of the *P. duryi* mitogenome is identical to that of the other species in GenBank, including *Biomphalaria* snails, close relatives within the family Planorbidae (DeJong et al. 2004). Additionally, phylogenetic analyses confirmed *P. duryi* as a sister species to *B. glabrata*, within the family Planorbidae of the Hygrophila (Figure 1). The characterization of the mitogenome of *P. duryi* can help elucidate challenging phylogenetic reconstruction of Gastropoda and provide a genomic resource for continued research on snails and their role in transmission of the infectious disease schistosomiasis.

## References

[CIT0001] Bernt M, Donath A, Jühling F, Externbrink F, Florentz C, Fritzsch G, Pütz J, Middendorf M, Stadler PF 2013 MITOS: improved *de novo* metazoan mitochondrial genome annotation. Mol Phylogenet Evol. 69:313–319. 22982435 10.1016/j.ympev.2012.08.023

[CIT0002] DeJong RJ, Emery AM, Adema CM 2004 The mitochondrial genome of *Biomphalaria glabrata* (Gastropoda: Basommatophora), intermediate host of *Schistosoma mansoni*. J Parasitol. 90:991–997. 15562597 10.1645/GE-284R

[CIT0003] Hahn C, Bachmann L, Chevreux B 2013 Reconstructing mitochondrial genomes directly from genomic next-generation sequencing reads—a baiting and iterative mapping approach. Nuc Acids Res. 41:e129. 10.1093/nar/gkt371 PMC3711436 23661685

[CIT0004] Jannotti-Passos LK, Ruiz JC, Caldeira RL, Murta SM, Coelho PM, Carvalho OS 2010 Phylogenetic analysis of *Biomphalaria tenagophila* (Orbigny, 1835) (Mollusca: Gastropoda). Mem Inst Oswaldo Cruz. 105:504–511. 20721500 10.1590/s0074-02762010000400027

[CIT0005] Kumar S, Stecher G, Tamura K 2016 MEGA7: Molecular evolutionary genetics analysis version 7.0 for bigger datasets. Mol Biol Evol. 33:1870–1874. 27004904 10.1093/molbev/msw054 PMC8210823

[CIT0006] Larkin MA, Blackshields G, Brown NP, Chenna R, McGettigan PA, McWilliam H, Valentin F, Wallace IM, Wilm A, Lopez R, et al. 2007 Clustal W and Clustal X version 2.0. Bioinformatics. 23:2947–2948. 17846036 10.1093/bioinformatics/btm404

[CIT0007] Nolan JR, Bergthorsson U, Adema CM 2014 *Physella acuta*: atypical mitochondrial gene order among panpulmonates (Gastropoda). J Molluscan Stud. 80:388–399. 25368439 10.1093/mollus/eyu025 PMC4214460

[CIT0008] Oskars TR, Bouchet P, Malaquias MA 2015 A new phylogeny of the Cephalaspidea (Gastropoda: Heterobranchia) based on expanded taxon sampling and gene markers. Mol Phylogenet Evol. 89:130–150. 25916189 10.1016/j.ympev.2015.04.011

[CIT0009] White TR, Conrad MM, Tseng R, Balayan S, Golding R, de Frias Martins AM, Dayrat BA 2011 Ten new complete mitochondrial genomes of pulmonates (Mollusca: Gastropoda) and their impact on phylogenetic relationships. BMC Evol Biol. 11:295. 21985526 10.1186/1471-2148-11-295 PMC3198971

[CIT0010] Williams ST, Foster PG, LIttlewood DT 2014 The complete mitochondrial genome of a turbinid vetigastropod from MiSeq Illumina sequencing of genomic DNA and steps towards a resolved gastropod phylogeny. Gene. 533:38–47. 24120625 10.1016/j.gene.2013.10.005

[CIT0011] Winnepenninckx B, Backeljau T, De Wachter R 1993 Extraction of high molecular weight DNA from molluscs. Trends Genet. 9:407. 8122306 10.1016/0168-9525(93)90102-n

[CIT0012] Zhang S-M, Bu L, Laidemitt MR, Lu L, Mutuku MW, Mkoji GM, Loker ES 2018 Complete mitochondrial and rDNA complex sequences of important vector species of *Biomphalaria*, obligatory hosts of the human-infecting blood fluke, *Schistosoma mansoni*. Sci Rep. 8:7341. 29743617 10.1038/s41598-018-25463-z PMC5943310

